# Malaria prevalence and transmission in the Zakpota sub-district of central Benin: baseline characteristics for a community randomised trial of a new insecticide for indoor residual spraying

**DOI:** 10.1186/s13071-024-06342-1

**Published:** 2024-07-13

**Authors:** Renaud Govoetchan, Augustin Fongnikin, Corneille Hueha, Juniace Ahoga, Chantal Boko, Thomas Syme, Riliwanou Issiakou, Abel Agbevo, Rock Aikpon, Graham Small, Janneke Snetselaar, Razaki Ossè, Filemon Tokponnon, Germain Gil Padonou, Corine Ngufor

**Affiliations:** 1 London School of Hygiene and Tropical Medicine (LSHTM), London, WC1E 7HT UK; 2 Centre de Recherche Entomologique de Cotonou (CREC), Cotonou, Benin; 3 Pan-African Malaria Vector Research Consortium (PAMVERC), Cotonou, Benin; 4 National Malaria Control Programme, Ministry of Health, Cotonou, Benin; 5 Innovative Vector Control Consortium (IVCC), Liverpool, UK

**Keywords:** Zakpota, Malaria prevalence, Vector density, *Anopheles*, Malaria transmission, Entomological inoculation rate

## Abstract

**Background:**

Malaria transmission is known to be perennial and heterogeneous in Benin. Studies assessing local malaria prevalence, transmission levels and vector characteristics are critical for designing, monitoring and evaluating new vector control interventions in community trials. We conducted a study in the Zakpota sub-district of central Benin to collect baseline data on household characteristics, malaria prevalence, vector characteristics and transmission dynamics in preparation for a randomised controlled trial to evaluate the community impact of VECTRON™ T500, a new broflanilide indoor residual spraying (IRS) product.

**Methods:**

A total of 480 children under 5 years of age from the 15 villages of the sub-district were tested for malaria by rapid diagnostic tests (RDTs). Mosquitoes were collected by human landing catches (HLCs), pyrethrum spray catches (PSCs) and Centers for Disease Control and Prevention miniature light traps (CDC-LTs) in selected houses in each village to assess vector density, composition, vector infectivity and prevalence of insecticide resistance markers. Bioassays were performed to detect vector susceptibility to pyrethroids, broflanilide (6 µg/bottle) and clothianidin (90 µg/bottle).

**Results:**

A total of 9080 households were enumerated in the 15 study villages. Insecticide-treated net (ITN) usage was > 90%, with 1–2 ITNs owned per household. Houses were constructed mainly with cement (44%) and mud (38%) substrates or a mixture of cement and mud (18%), and 60% of them had open eaves. The overall prevalence of *P. falciparum* infection was 19% among surveyed children: 20% among females and 18% among males. The haemoglobin rate showed an anaemia (< 11 g/dl) prevalence of 66%. *Anopheles coluzzii* and *An. gambiae* sensu stricto (s.s.) were the two vector species present at an overall proportion of 46% versus 54%, respectively. The human biting rate was 2.3 bites per person per night (b/p/n) and biting occurred mostly indoors compared with outdoors (IRR = 0.776; *P* = 0.001). The overall proportion of outdoor biting was 44% and exceeded indoor biting in three villages. The sporozoite rate was 2% with a combined yearly entomological inoculation rate (EIR) of 16.1 infected bites per person per year (ib/p/y). There was great variability in malaria transmission risk across the villages, with EIR ranging from 0 to 29.3 ib/p/y. The vector population showed a high intensity of resistance to pyrethroids across the study villages but was largely susceptible to broflanilide and clothianidin.

**Conclusions:**

This study found high levels of malaria prevalence, vector density and transmission in the Zakpota sub-district despite the wide use of insecticide-treated nets. The vector population was mostly indoor resting and showed a high intensity of pyrethroid resistance but was generally fully susceptible to broflanilide. These findings demonstrated the suitability of the study area for the assessment of VECTRON™ T500 in a community randomised trial.

**Graphical Abstract:**

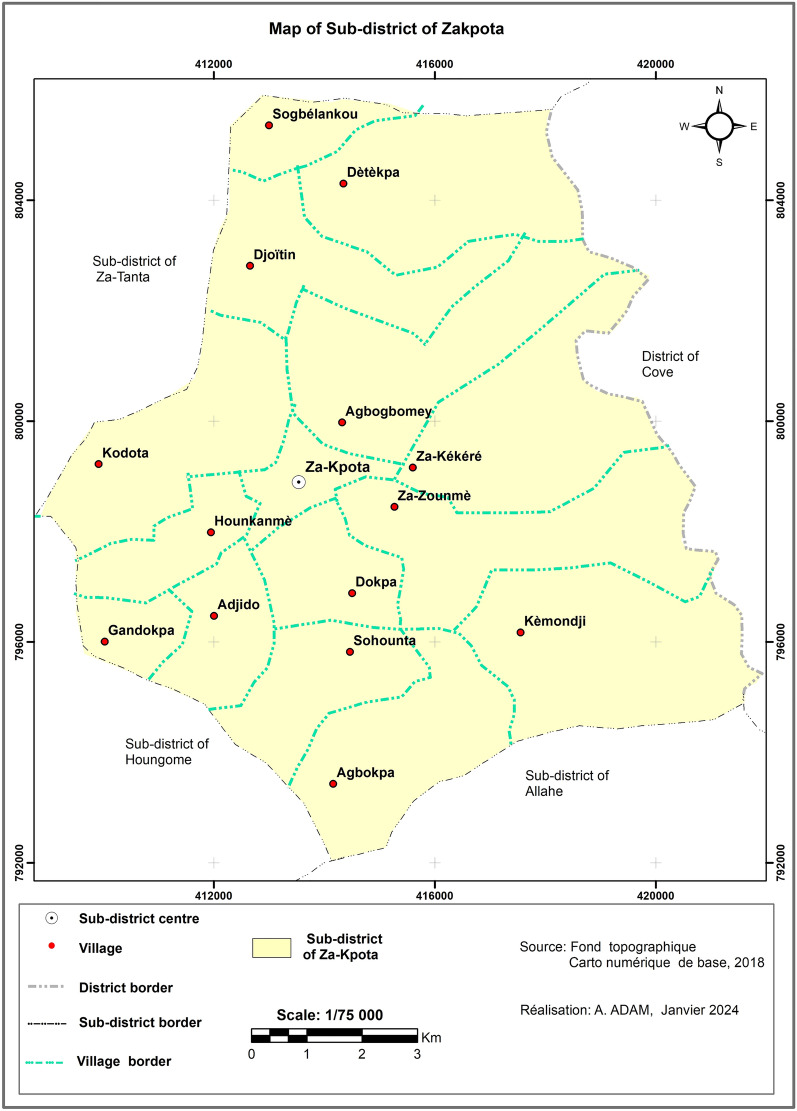

**Supplementary Information:**

The online version contains supplementary material available at 10.1186/s13071-024-06342-1.

## Background

Despite considerable worldwide efforts deployed in recent years to control malaria, the disease remains a major public health problem in many endemic countries [1, 2]. Malaria cases continue to rise globally, reaching 249 million in 2022, with the African Region shouldering about 95% of cases and 96% of deaths [1]. As in most sub-Saharan countries, malaria remains a serious public health challenge in the Republic of Benin, accounting for 50% of all medical consultations, 40% of hospital admissions in health facilities and 20% of deaths among children under 5 years in 2021 [3].

While malaria transmission is known to be perennial in Benin, there are reports of heterogeneity between regions [4–6]. A cross-sectional epidemiological and clinical survey involving  > 10,000 children from 72 villages in two health districts showed that the prevalence of *Plasmodium falciparum* infection was moderate in the south and high in the north, with more heterogeneity in the southern parts of the country [7]. A recent transmission intensity stratification study using entomological inoculation rates (EIRs) also demonstrated great variability across districts and regions [8]. While mosquitoes of the *Anopheles gambiae* complex (mostly *An. gambiae* and *An. coluzzii)* play a major role in the transmission of *P. falciparum* from the north-to-south transect of the country [8, 9], *An. funestus* [10–12] and *An. nili* have also been implicated as secondary vectors [11, 13]. *Anopheles gambiae* and *An. coluzzii* exhibit broadly similar anthropophagic and indoor biting behaviour in Benin, with the latter showing a predominant presence during the dry seasons while the former is more abundant in wet seasons [8, 9, 14, 15]. Pyrethroid resistance, mediated by the L1014F mutation and overexpressed P450 enzymes, is pervasive in *Anopheles* vector mosquitoes in Benin and of moderate to high intensity across the different ecological zones of the country [16–18]. Resistance to organophosphate and carbamate insecticides has been reported in some areas in Benin [19, 20] but is less prevalent than pyrethroid resistance [15, 17, 18, 21–24].

Malaria prevention and control in Benin is largely dependent on the large-scale deployment of insecticide-treated nets (ITNs) via mass campaigns. However, studies have reported a persistent high malaria prevalence in many regions of Benin despite high ITN coverage and use [4, 25] and this has been attributed to high levels of pyrethroid resistance in local vectors and poor durability of ITNs. The identification of novel active ingredients (AIs) that can be used on ITNs and for indoor residual spraying (IRS) for effective control of pyrethroid-resistant malaria vectors is a priority for the vector control community [26, 27]. This requires cluster randomised trials to investigate their impact under community use in different epidemiological settings. Assessing local malaria prevalence, transmission levels and vector characteristics is critical for designing, monitoring and evaluating new vector control interventions [28, 29] in community trials given the heterogeneity in transmission and high variability in vector abundance and composition in most endemic settings [30]. Such data might also be useful for decision-making for the deployment of appropriate context-specific vector control interventions. The present study was conducted in the Zakpota sub-district of central Benin to collect baseline data on malaria prevalence, vector characteristics and transmission dynamics in preparation for a randomised controlled trial to evaluate the community impact of the newly developed VECTRON™ T500, a novel broflanilide insecticide product for IRS [31]. The AI is a meta-diamide that acts as a non-competitive antagonist of the γ-aminobutyric acid (GABA) receptor of chloride channels of the insect inhibitory nervous system thus presenting a new mode of action for malaria vector control [32]. The randomised controlled trial evaluated the efficacy and residual activity of VECTRON™ T500 compared to Fludora^®^ Fusion, a World Health Organization prequalification (WHO/PQ)-listed wettable powder IRS formulation of a mixture of clothianidin and deltamethrin.

## Methods

### Study area

**Fig. 1:**
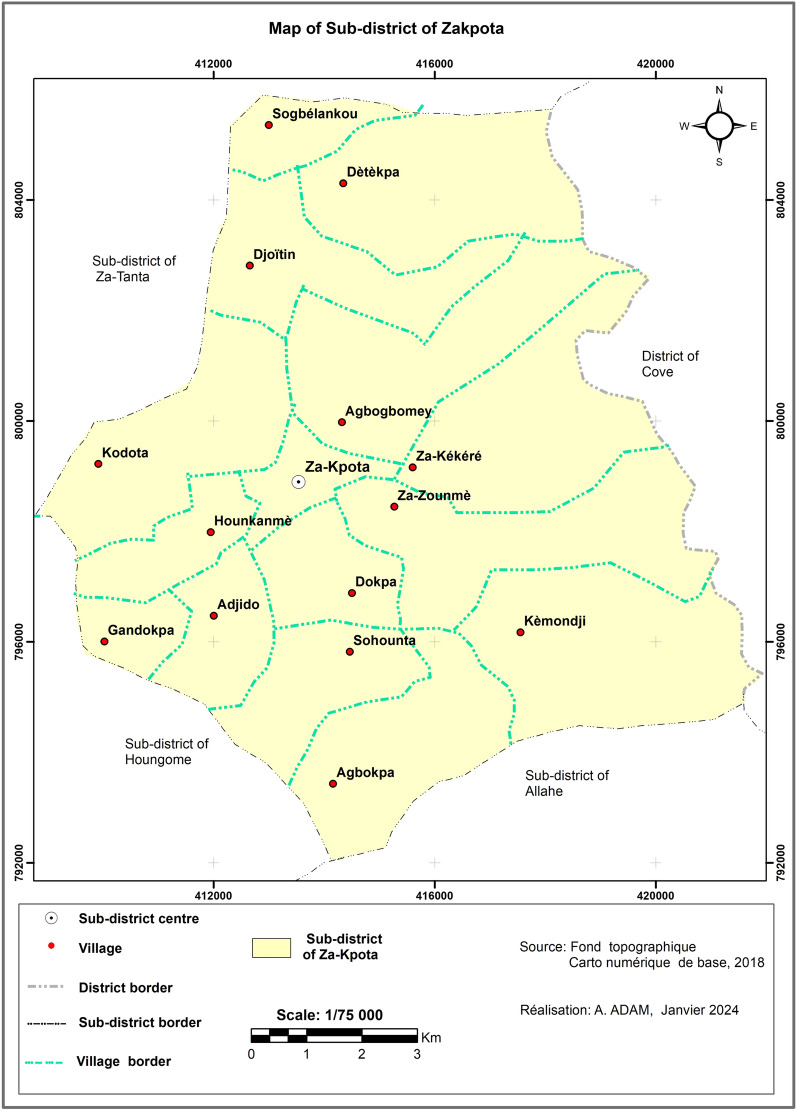
Spatial distribution of the 15 villages in the sub-district of Zakpota

The study was conducted in the Zakpota Centre sub-district located in the department of Zou, southern Benin. The study area covers 600 km^2^ separated into 15 administrative villages (Fig. 1). Economic activities of the population include agriculture (85%), trade (8%), crafts (5%) and other services (2%) [33]. The Zakpota Centre sub-district has a sub-equatorial climate with two rainy seasons (April–July and September–November) and two dry seasons (December–March and July–August). The average annual rainfall of Zakpota is 980 mm, with important inter-annual fluctuations over the past 40 years, a source of climatic uncertainty for this area. Malaria prevention in Zakpota mainly relies on the distribution of ITNs through mass campaigns led by the Ministry of Health. This is supplemented by routine distributions to pregnant women and children under 5 years through antenatal visits and the expanded programme of immunisation.

### Household survey

Detailed information on the distribution, location, number, type, size and accessibility of households was needed for the IRS campaign. Mapping and censuses were conducted in November 2020 to generate a database of all houses in the study area. Every building of each hamlet/village, village boundaries, health facilities and other landmarks were geo-referenced, mapped and recorded in the field using integrated handheld tablets fitted with global positioning system (GPS). Information was collected on the size of the household, house structure, the number of rooms, availability and type of bednets/ITNs, number of sleeping places, and wall substrate type in each village using a structured questionnaire.

### Cross-sectional clinical survey

A cross-sectional clinical survey was conducted in May 2021 to obtain data on malaria prevalence and anaemia in children under 5 years of age in all 15 villages in the Zakpota sub-district, before the implementation of the IRS campaign. The survey team included experienced nurses and community health workers (CHWs). A sample size of 480 participants was found sufficient based on sample size calculations performed using a baseline malaria prevalence of 46% observed in a recent survey conducted in the district [34] and a coefficient of variation of 0.3. Using the census data, a random selection of 30 households with children under 5 years were visited in each of the villages where one child was enrolled in the study per household giving a total of 480 study participants. Participating children were tested for malaria using a rapid diagnostic test (RDT) (CareStart malaria HRP2/pLDH [Pf/pan] combo, DiaSys, UK) irrespective of symptoms. Each child's axillary temperature was assessed, and those who tested positive by RDT and showed a fever (> 37.5 °C) were considered symptomatic for malaria and were provided free treatment. The haemoglobin concentration (g/dl)xxx was measured using a Hemo Control diagnostic analyser (EKF Diagnostics, Cardiff, UK) to assess anaemia in all enrolled children. Children having a haemoglobin level below 11 g/dl were considered anaemic.

### Entomological data collection

Baseline entomological studies were conducted every 2 months between August 2020 and April 2021 over four sampling rounds in the 15 villages of the Zakpota Centre sub-district. Data on the presence, distribution, behaviour, age structure, insecticide susceptibility status of vectors and baseline vector parameters were collected in each village of the study area. Mosquitoes were collected using three methods.

- Human landing catches (HLCs) were performed for two nights at each time point, in six randomly selected houses in each village. Four consenting human volunteer mosquito collectors were used per house, with volunteers working in pairs—one collecting mosquitoes indoors and the other outdoors. Collections were performed between 07:00 p.m. and 07:00 a.m., with pairs alternating every 6 h, i.e. 07:00 p.m. to 01:00 a.m. and 01:00 a.m. to 07:00 a.m. Collectors sat on chairs indoors and outdoors with their legs exposed; the outdoor collector was positioned at least 10 m from the house. Using flashlights, collectors caught mosquitoes landing on their legs with haemolysis tubes, and each hour’s collection was kept separately.
- Centers for Disease Control and Prevention miniature light traps (CDC-LTs) (Model 512, John W. Hock, FL, USA) were used to collect mosquitoes for two nights at every time point inside six randomly selected houses per village. The traps were set up daily at approximately 1–1.5 m above the ground and close to human-occupied untreated bednets. The light traps were operated overnight between 7:00 p.m. and 7:00 a.m. daily.
- Pyrethrum spray catches (PSCs) were also performed in six randomly selected houses in each village. On each day of sampling, all food and water were removed from the house before spraying and white sheets were laid on the floor. The house was sprayed with an aerosol (Rambo^@^) containing 0.25% transfluthrin and 0.20% permethrin. All mosquitoes falling onto the white sheets 15 min post-spraying were collected using forceps.

### Processing of mosquito samples

Mosquitoes were identified to species level using morphological keys [35]. *Anopheles gambiae* sensu lato (s.l.) sibling species identification was performed using the polymerase chain reaction (PCR) method [36]. For all sampling methods, the head and thorax of each mosquito collected were separated from the abdomen and tested for the presence of *P. falciparum* circumsporozoite protein (CSP) using direct enzyme-linked immunosorbent assay (ELISA) [37]. Sporozoite rates (SRs) for each village were estimated as the proportion of *P. falciparum*-positive mosquitoes, while the EIRs were estimated as the number of infective bites per person per night. The abdomens of unfed mosquitoes from all three collections morphologically identified as *Anopheles gambiae* s.l. were dissected to assess parity using Detinova’s ovary tracheation method [38]. The parity rate was measured as the ratio of parous mosquitoes out of the total number dissected. A sub-sample of mosquitoes morphologically identified as *An. gambiae* s.l. was processed for the presence of the L1014F and G119S insecticide resistance mutations using the methods described by Martinez-Torres [39] and Weill [40], respectively.

### Insecticide susceptibility monitoring

WHO bottle bioassays were used to assess the susceptibility of mosquitoes from each village to clothianidin and broflanilide, the AIs contained respectively in Fludora^®^ Fusion and VECTRON™ T500, the IRS products evaluated in the RCT after the present baseline data collections. Clothianidin was tested at a discriminating concentration of 90 µg/bottle provided by the manufacturer while broflanilide was tested at 6 µg/bottle determined in preliminary dose–response bioassays [41]. To prevent crystallization of the insecticide in bottle bioassays and to improve bioavailability and uptake by exposed mosquitoes, Mero^®^ (81% rapeseed oil methyl ester; Bayer CropScience) was used as an adjuvant at a dose of 500 parts per million (ppm) for broflanilide and 1000 ppm for clothianidin. The intensity of resistance to deltamethrin in each study arm was also assessed at baseline in bottles treated at 1×, 2×, 5× and 10× the discriminating concentration of 12.5 µg/bottle. One hundred unfed wild adult female first filial generation (F1) *An. gambiae* s.l. (2–5 days old) raised in insectary from larvae collected from breeding sites in study villages were exposed for 1 h in bottles treated with insecticide in batches of 25 mosquitoes per bottle. After exposure, the mosquitoes were gently aspirated from the bottle into clean, labelled paper cups and provided with 10% sugar solution-soaked cotton wool. Knockdown was recorded after exposure, and mortality was recorded 24 h after exposure to deltamethrin, 72 h after exposure to broflanilide and 120 h after exposure to clothianidin due to the different modes of action and speed of kill of these insecticides. Mosquitoes were also exposed in bottles treated with acetone and Mero alone to serve as controls.

### Ethical considerations

This study was approved by the national ethics review committee of the Ministry of Health Benin (CNERS, No. 29 and 51) and the ethics review board of the London School of Hygiene & Tropical Medicine (LSHTM, Ref: 22453). Before any project activities, village and hamlet leaders were invited to sensitization sessions and written informed consent was obtained from them before starting data collection. Heads of households involved in the study gave informed consent before participating in the study. Written informed consent was obtained from parents of children enrolled in the clinical survey. Any children found positive for malaria were provided free anti-malaria treatment. Mosquito collectors were trained and they also provided written informed consent. All participating households and individuals were free to withdraw from the studies at any time.

### Data management and analysis

The census and prevalence survey data were captured on electronic forms using smartphones installed with OpenDataKit (ODK) Collect. Entomological data was collected on pre-designed data collection forms and then double-entered into pre-established Microsoft Excel databases. All data were stored on a secure server located at the Centre de Recherche Entomologique de Cotonou (CREC)/LSHTM Facility in Benin and made available for statistical analysis. Descriptive statistics were used to summarise demographic data. Differences in malaria prevalence were analysed using mixed-effects logistic regression models, while differences in indoor and outdoor vector density were analysed using mixed-effects negative binomial regression. All analyses were performed with Stata version 17 (StataCorp LLC).

The resistance status of wild vector populations of *An. gambiae* s.l. was interpreted according to WHO guidelines [42, 43]: mortality ≥ 98% indicates susceptibility, mortality less than 90% indicates resistance, and mortality of 90–97% means a possible resistance that needs to be confirmed. The intensity of resistance was interpreted as low, moderate or high intensity when 24-h mortality rates were ≥ 98% at 5× the discriminating concentration, ≥ 98% at 10× the discriminating concentration or < 98% at 10× the discriminating concentration, respectively.

## Results

### Characteristics of study population and households

Summary data collected on demography and housing structure characteristics during the baseline census are presented in Tables 1 and 2, respectively. Overall, 34,976 people living in 9080 households were enumerated in the 15 study villages of which 48% were females. The mean number of persons per household was 3–5 with 1–2 ITNs owned per household. All ITNs found in the study area were treated only with pyrethroids and the predominant brands were Yorkool^®^ and PermaNet^®^ 2.0 distributed in the 2020 mass campaign by the Ministry of Health. The proportion of the population under 5 years old was 15%, while pregnant women constituted 2% of the population (Table 1).

**Table 1:** Demographic data in the 15 administrative villages of the sub-district of Zakpota Centre

	Gender				Malaria-vulnerable population				Household					
	Male	%	Female	%	Pregnant women	%	Children under 5 years	%	Total	Mean number of people per HH	Mean number of sleeping places	Mean number of sleeping places	Mean number ITNs	Mean number ITNs
Adjido	1630	53	1470	47	45	1	437	14	903	3	1006	1	1090	1
Agbogbomey	835	51	808	49	48	3	218	13	417	4	756	2	626	2
Agbokpa	741	53	665	47	22	2	227	16	368	4	597	2	559	2
Detekpa	733	51	707	49	21	1	194	13	398	4	621	2	641	2
Djoyitin	1031	52	956	48	42	2	319	16	554	4	886	2	923	2
Dokpa	618	49	649	51	21	2	206	16	263	5	533	2	501	2
Gnandokpo	408	53	367	47	28	4	106	14	187	4	303	2	289	2
Hounkanme	1408	53	1242	47	73	3	427	16	658	4	1026	2	944	1
Kemondji	1173	53	1059	47	57	3	375	17	473	5	949	2	882	2
Kodota	1479	51	1406	49	66	2	432	15	638	5	1391	2	1144	2
Sogbelankou	858	52	788	48	33	2	229	14	484	3	622	1	746	2
Sohounta	898	53	792	47	55	3	310	18	429	4	693	2	702	2
Za-Kekere	1775	50	1750	50	75	2	524	15	852	4	1574	2	1703	2
Za-Kpota Centre	3737	51	3601	49	173	2	1112	15	1978	4	3351	2	2993	2
Za-Zounme	712	51	680	49	26	2	209	15	478	3	631	1	656	1
Total	18,036	52	16,940	48	785	2	5325	15	9080	4	14,939	2	14,399	2

**Table 2:** Housing structure characteristics in the 15 administrative villages of the Zakpota Centre sub-district

	Total	% Eligible household (95%CI)	Substrate of inner wall			Aspect of inner wall			Eave	
			% Cement (95%CI)	% Mud (95%CI)	% Cement & Mud (95%CI)	% Plastered (95%CI)	% Not plastered (95%CI)	% Partially plastered (95%CI)	% Close (95%CI)	% Open (95%CI)
Adjido	1216	88 (86–90)	37 (34–41)	42 (39–45)	21 (19–23)	49 (46–52)	40 (37–43)	11 (9–13)	29 (26–32)	71 (68–74)
Agbogbomey	578	90 (88–92)	55 (51–59)	26 (22–30)	19 (16–22)	57 (54–60)	32 (28–36)	11 (8–14)	54 (50–58)	46 (42–50)
Agbokpa	570	86 (83–89)	15 (12–18)	79 (76–82)	6 (4–8)	20 (17–23)	77 (74–80)	3 (2–4)	37 (33–41)	63 (59–67)
Detekpa	554	83 (80–86)	29 (25–34)	58 (54–62)	13 (9–15)	39 (35–43)	54 (50–58)	7 (5–9)	19 (16–22)	81 (78–84)
Djoyitin	713	83 (80–86)	39 (35–44)	45 (41–49)	16 (13–19)	43 (39–47)	54 (50–58)	3 (2–4)	39 (35–43)	61 (57–65)
Dokpa	418	92 (89–95)	40 (35–45)	49 (44–54)	11 (8–14)	52 (47–57)	37 (32–42)	11 (9–13)	34 (29–39)	66 (61–71)
Gnandokpo	254	89 (85–93)	33 (27–39)	46 (40–52)	21 (16–26)	45 (39–51)	51 (45–56)	4 (2–6)	14 (10–18)	86 (82–90)
Hounkanme	977	86 (84–88)	50 (47–53)	32 (29–35)	18 (16–20)	58 (55–61)	37 (34–40)	5 (4–7)	46 (43–49)	54 (51–57)
Kemondji	808	86 (84–88)	28 (25–31)	59 (56–62)	13 (10–14)	34 (31–37)	63 (60–66)	3 (2–4)	26 (23–29)	74 (71–77)
Kodota	935	93 (91–95)	49 (46–52)	41 (38–44)	10 (9–13)	68 (65–71)	27 (24–30)	5 (4–6)	39 (36–42)	61 (58–64)
Sogbelankou	674	81 (78–84)	29 (26–32)	53 (49–57)	18 (14–20)	36 (32–40)	61 (57–65)	3 (1–4)	30 (27–33)	70 (67–73)
Sohounta	646	93 (91–95)	45 (41–49)	36 (32–40)	19 (15–21)	54 (50–58)	44 (40–48)	2 (1–3)	42 (38–46)	58 (54–62)
Za-Kekere	1391	87 (85–89)	55 (52–58)	27 (25–29)	17 (15–19)	49 (46–52)	47 (44–50)	4 (3–5)	51 (48–54)	49 (46–52)
Za-Kpota Centre	2325	90 (89–91)	58 (56–60)	18 (16–20)	24 (22–26)	66 (64–68)	23 (21–25)	11 (10–12)	50 (48–52)	50 (48–52)
Za-Zounme	549	89 (86–92)	35 (30–38)	36 (32–40)	29 (27–31)	47 (43–51)	44 (40–48)	9 (7–11)	38 (34–42)	62 (58–66)
Total	12,608	88 (87–89)	44 (43–45)	38 (37–39)	18 (17–19)	51 (50–52)	42 (41–43)	7 (6–8)	40 (39–41)	60 (59–61)

A total of 12,608 house structures were enumerated across the 15 villages and 88% of them were eligible for IRS (range 81% to 93% per village) as defined by WHO guidelines [44]. The houses were constructed mainly with cement (44%) or mud (38%) substrates while some were constructed with a mixture of cement and mud (18%). Overall, 51% of surveyed structures were plastered and 60% had open eaves (Table 2).

### Malaria prevalence results

A total of 480 children under the age of 5 living in the sub-district of Zakpota Centre were surveyed of which 51% were female (Table 3). The age structure was broadly similar between both genders with children < 2 years constituting 26% of the sample size. ITN usage was very high amongst the children tested (> 90%) and similar between genders. The proportion of children reporting fever within the last 48 h was 17% among both males and females (Table 4).

**Table 3:** Characteristics and age structure of children under 5 years tested in the malaria prevalence survey in the sub-district of Zakpota Centre

	Female		Male		Total	
	*N*	% (95% CI)	*N*	% (95% CI)	*N*	% (95% CI)
Number of children	244	51 (47–55)	236	49 (45–43)	480	100
Age structure (year)						
0–1	3	1 (0–2)	1	0 (0–1)	4	1 (0–2)
1–2	61	13 (10–16)	58	12 (9–15)	119	25 (21–29)
2–3	68	14 (11–17)	65	14 (11–17)	133	28 (24–32)
3–4	50	10 (7–13)	44	9 (6–12)	94	20 (16–24)
4–5	62	13 (10–16)	68	14 (11–17)	130	27 (23–31)
Slept under net (%)	220	90 (87–93)	212	90 (87–93)	432	90 (87–93)
Fever within last 48 h	42	17 (14–20)	39	17 (14–20)	81	17 (14–20)

**Table 4:** Axillary fever, haemoglobin rate and malaria prevalence results in children under 5 years in the sub-district of Zakpota Centre

	Female		Male		Total	
	*N*	% (95% CI)	*N*	% (95% CI)	*N*	% (95% CI)
Number of children	244	51 (47–55)	236	49 (45–43)	480	100
Axillary temperature						
< 37.5 °C	235	96 (94–98)	222	94 (91–97)	457	95 (93–97)
≥ 37.5°c	9	4 (2–6)	14	6 (3–9)	23	5 (3–7)
Haemoglobin rate						
< 11 g/dL	168	69 (63–75)	151	64 (58–70)	319	66 (62–70)
≥ 11 g/dL	76	31 (25–37)	85	36 (30–42)	161	34 (30–38)
RDT results (*P. falciparum*)						
Positive	50	20 (15–25)	43	18 (13–23)	93	19 (15–23)
Negative	194	80 (75–85)	193	82 (77–87)	387	81 (77–85)

### Impact of age and ITN ownership on malaria prevalence

Table 5 presents the ratio of geometric means of sub-district levels of *P. falciparum* infection rates for age groups and net ownership status. In the households surveyed, the infection rate was significantly lower in children < 2 years than in children aged 2–5 years (3% *vs* 16%, OR: 1.839, 95% CI 1.027–3.294, *P* = 0.04). The number of ITNs in the households appeared to have no association with the malaria infection rate in children. Infection rates were lower in households owning at least two ITNs compared to households owning at least one ITN (2% vs. 18% OR: 2.471, 95% CI 1.191–5.125, *P* = 0.015).

**Table 5:** Risk factors associated with *P. falciparum* infection in households

	Malaria infected household		RR* (95% CI)	*P*-value
	*N*	% (95% CI)		
Total of households	480	100	–	–
Age				
< 2 years	16	3 (1–5)	1.924 (0.954–3.880)	0.067
2–5 years	77	16 (13–19)		
Net ownership				
≤ 1 LN	7	2 (1–3)	2.022 (1.004–4.072)	0.05
≥ 2 LNs	86	18 (15–21)		

^a^Ratio of geometric means of village level *P. falciparum* infection rates

### Mosquito composition and biting times

**Fig. 2:**
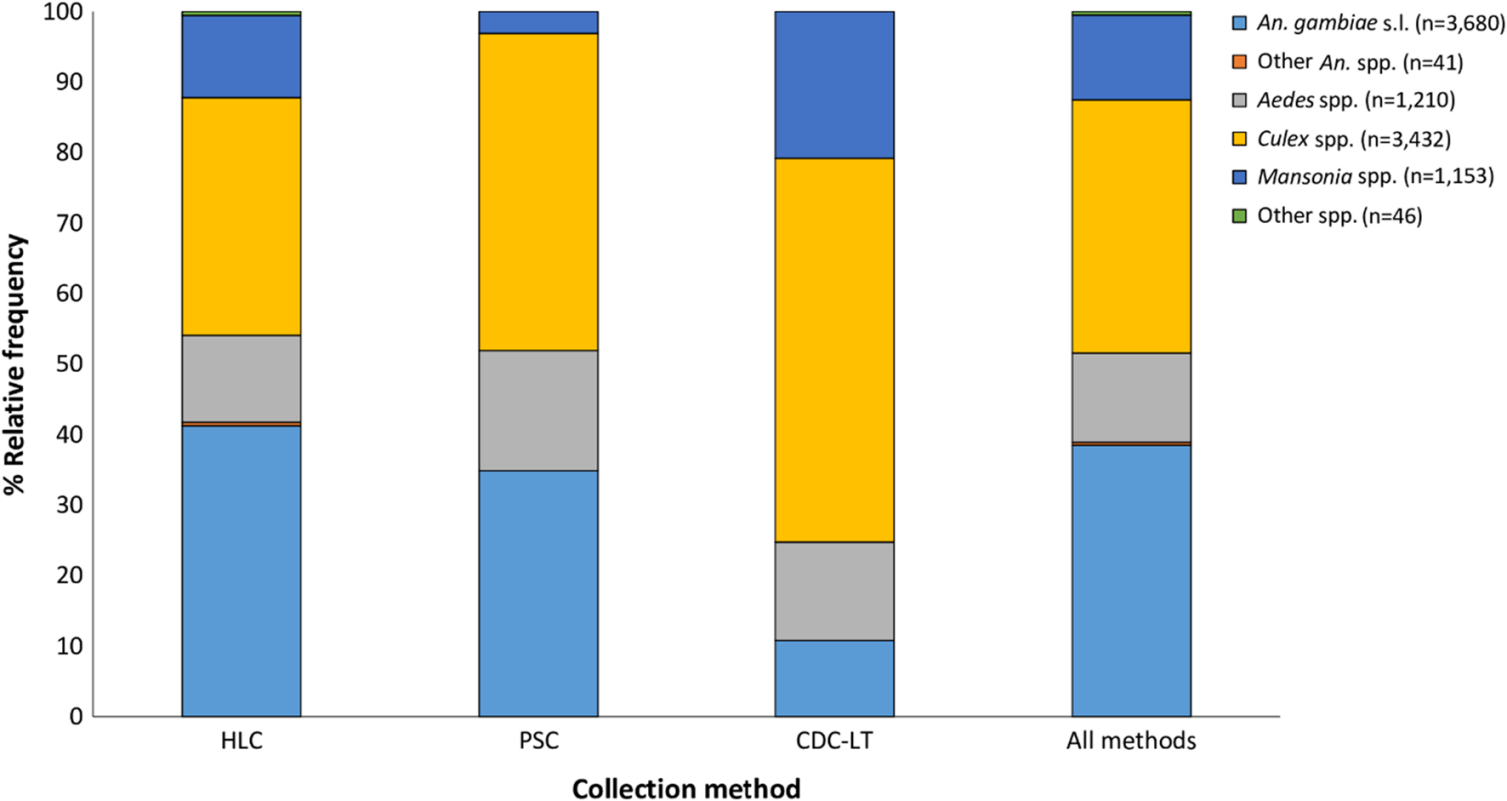
Overall mosquito species composition in the study area per sampling method

**Fig. 3:**
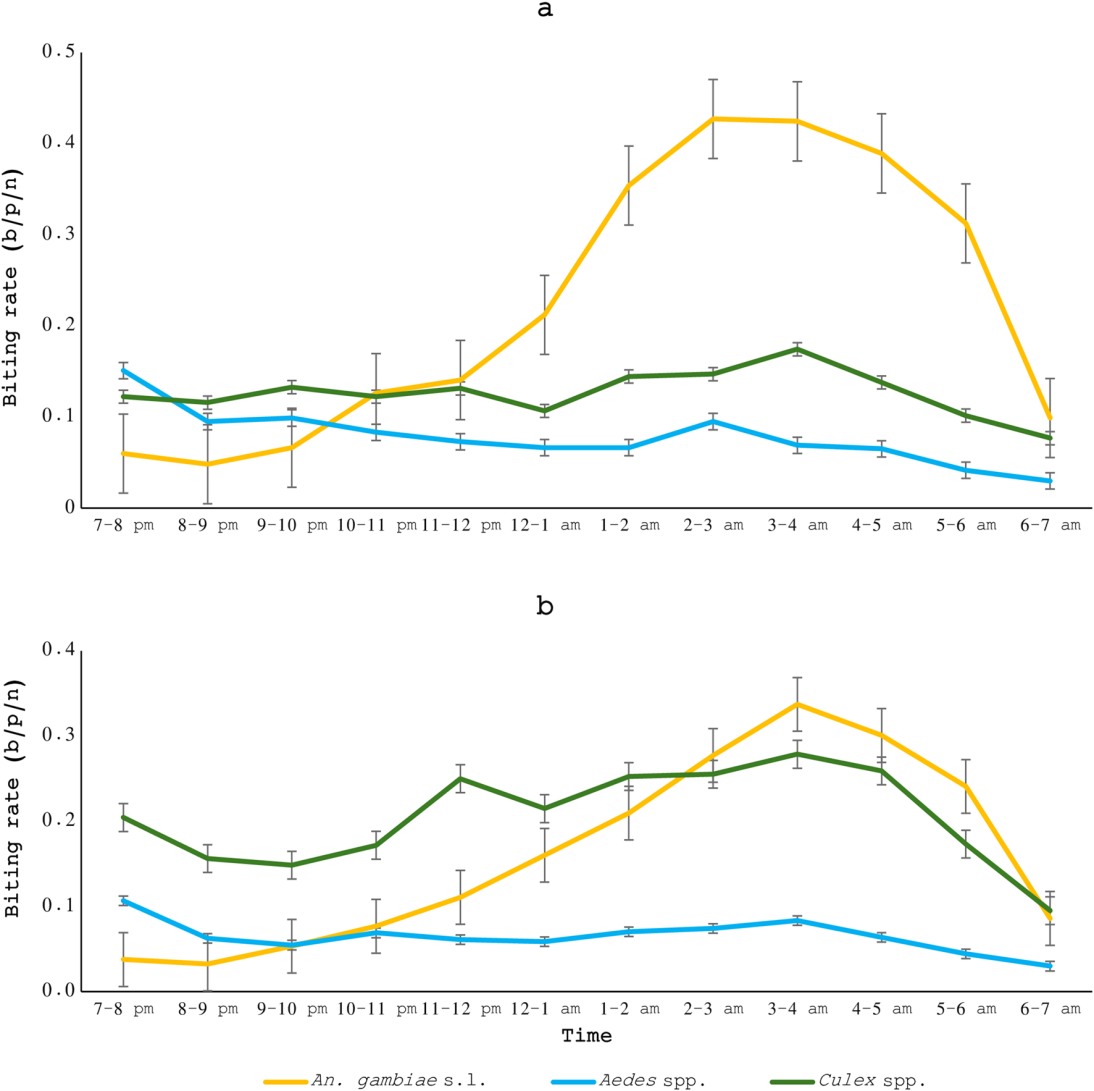
*An. gambiae* s.l., *Aedes* spp. and *Culex* spp. mosquitoes hourly indoor (**a**) and outdoor (**b**) biting rates in the study area

A total of 9562 female mosquitoes were collected in the study area during the four sampling time points using HLC, PSC and CDC-LT sampling methods. *Anopheles gambiae* s.l. was the predominant species, representing 38% of the cumulative collections for all three methods. Comparing mosquito composition between collection methods, the HLC was more efficient in collecting *An. gambiae* s.l. while the PSC and CDC-LT methods were more efficient in collecting *Culex* spp. mosquitoes (Fig. 2). All three collection methods collected approximately equal proportions of *Aedes* spp. mosquitoes (~ 13%). Peak mosquito biting times were 1:00 a.m. to 5:00 a.m. for *An. gambiae* s.l., early evening (before 8 p.m.) for *Aedes* spp., and 11:00 p.m. to 5:00 a.m. for *Culex* spp. (Fig. 3). Biting during peak hours was higher indoors for *An. gambiae* s.l. and *Aedes* spp., and outdoors for *Culex* spp. (Fig. 3).

### *Anopheles gambiae* sibling species composition

**Fig. 4:**
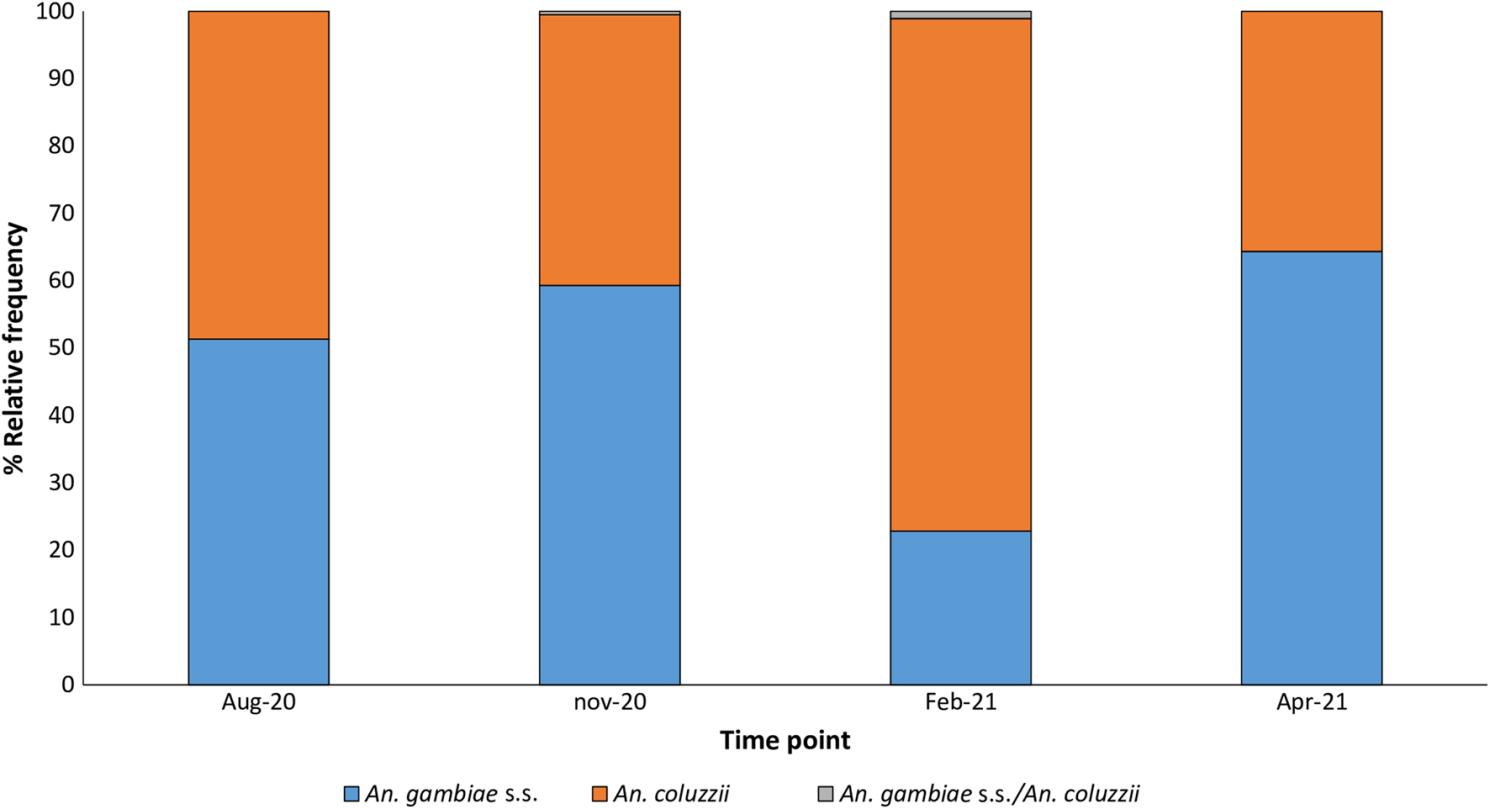
*An. gambiae* sibling species composition

A total of 3460 *An. gambiae s.l.* mosquitoes were collected and 95% of these were collected by HLCs. The CDC LT and PSC methods collected very small proportions of *An. gambiae s.l.* (5% for both methods). A total of 471 specimens of *An. gambiae s.l.* from the study area were analysed for sibling species identification. *An. coluzzii* and *An. gambiae s.s.* were the 2 species present at an overall proportion of 46% and 54% respectively. The proportions of each species fluctuated depending on the collection time points with *An. gambiae s.s.* being more abundant at most time points (52–64%) except in February 2021 (Fig. 4).

### Vector density indoors and outdoors

**Fig. 5:**
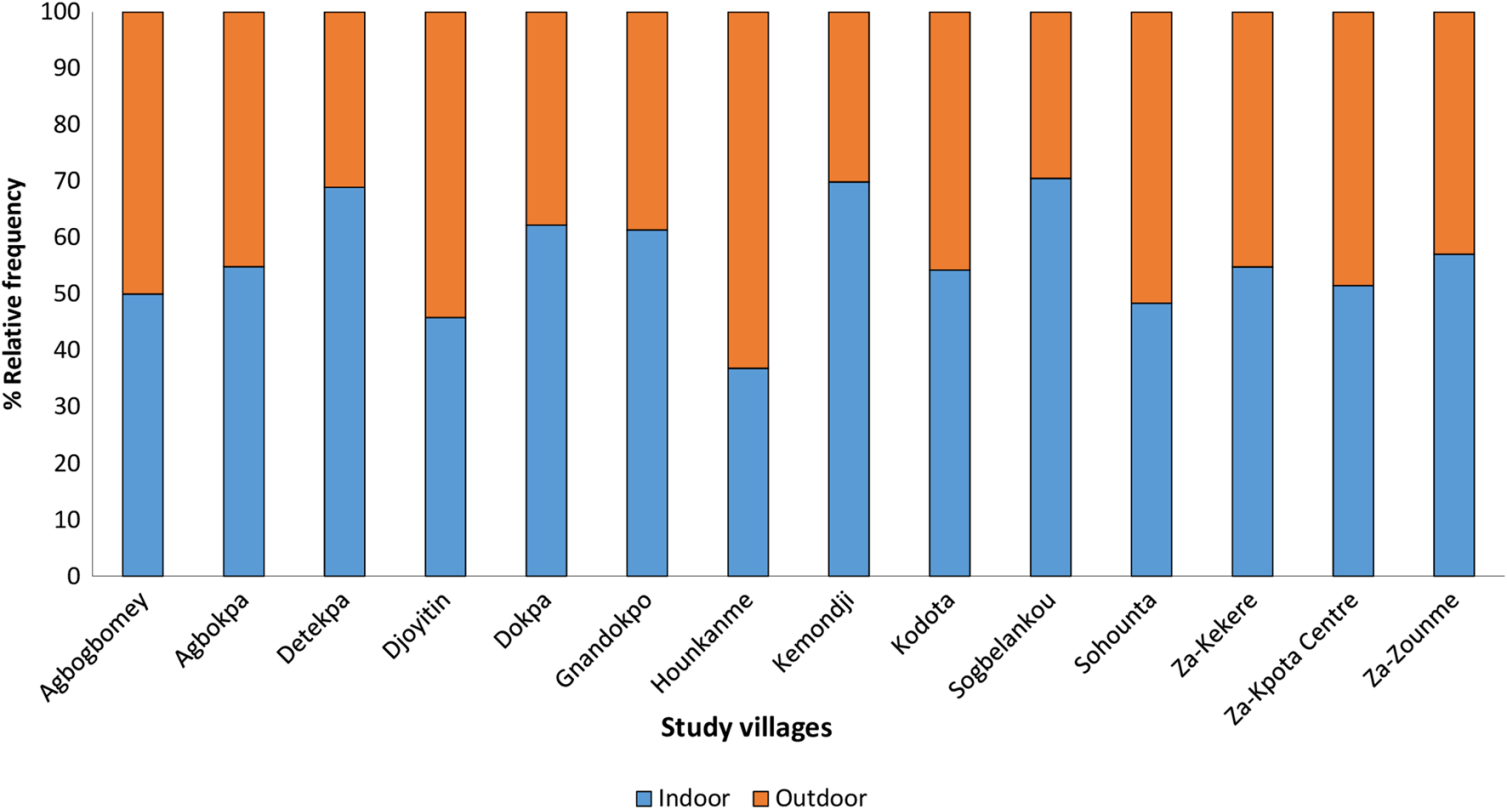
Indoor and outdoor density of *An. gambiae s.l.* in the study villages of the sub-district of Zakpota Centre

A total of 3460 *An. gambiae s.l.* vector mosquitoes were collected during the study of which 58% were collected indoors and 42% outdoors. The aggregated indoor density of *An. gambiae s.l.* was significantly higher than outdoor (incidence rate ratio (IRR) = 0.776; *p* = 0.001). Vector densities were higher indoors compared to outdoors in most villages (12 out of 15) except in Djoyitin, Hounkanme and Sohounta where outdoor biting was higher (Fig. 5).

### Human biting rate and infectivity of *An. gambiae* s.l.

Table 6 presents the human biting rate (HBR), SR and yearly EIR in *An. gambiae* s.l. across the study villages. The overall mean HBR of the study area was 2.3 bites per person per night (b/p/n), and the range between villages was 0.3 b/p/n (Agbokpa) to 5.8 b/p/n (Za-Kekere) (Table 6). Mosquito infection rates determined by CSP ELISA performed on a total of 2863 *An. gambiae* s.l. mosquitoes showed a mean SR of 2%, with a village range of 0% in Sohounta to 8.5% in Kodota. Combined data revealed an overall yearly EIR of 16.1 infected bites per person per year (ib/p/y) in the study area. The villages with the highest malaria transmission risk were Agbogbomey (29.3 b/p/y), Za-Zakere (27.5 ib/p/y), Kemondji (25.7 ib/p/y), Detekpa (23.5 ib/p/y) and Za-Zounme (22.6 ib/p/y).

**Table 6:** Human biting rate, sporozoite rate and entomological inoculation rate of *An. gambiae* s.l. in the villages of the sub-district of Zakpota

	N collected^a^	Man.night	HBR (b/p/n)	Total analysed	SR (%)	EIR (ib/p/y)
Agbogbomey	340	96	3.5	309	2.3	29.3
Agbokpa	31	96	0.3	26	7.7	9.1
Detekpa	354	96	3.7	229	1.7	23.5
Djoyitin	277	192	1.4	230	1.7	9.2
Dokpa	180	96	1.9	168	3.0	20.4
Gnandokpa	40	96	0.4	40	7.5	11.4
Hounkanmey	38	96	0.4	37	0.0	0.0
Kemondji	312	96	3.3	277	2.2	25.7
Kodota	59	96	0.6	59	8.5	19.0
Sogbelankou	295	96	3.1	208	0.5	5.4
Sohounta	31	96	0.3	24	0.0	0.0
Za-Kekere	1109	192	5.8	919	1.3	27.5
Za-Kpota Centre	33	96	0.3	33	6.1	7.6
Za-Zoume	361	96	3.8	304	1.6	22.6
Total	3460	1536	2.3	2863	2.0	16.1

^*a*^Collected by HLC in four sampling time points

*HBR* human biting rate, *SR* sporozoite rate, *EIR* entomological inoculation rate

*man.night*: number of mosquito collectors x number of collection nights

### Parous rate in *An. gambiae* s.l.

Table 7 presents *An. gambiae* s.l. parous rates across study villages in the sub-district of Zakpota Centre. Of the 1145 specimens of *An. gambiae* s.l. dissected over the study period, 561 were parous representing 47%. The parous rate per village ranged from 17% (Sogbélankou) to 100% (Kodota).

**Table 7:** Parous rate in *An. gambiae* s.l. in the villages of sub-district of Zakpota Centre

Village	Total dissected	N Parous	Parous rate (%)	95% CI
Adjido	9	5	56	24–88
Agbogbome	141	73	52	44–60
Detekpa	172	48	28	21–35
Djoitin	49	22	45	31–49
Gnandokpa	13	8	62	36–88
Hounkanmey	9	6	67	36–98
Kemondji	68	42	62	50–74
Kodota	21	21	100	–
Sogbelankou	127	21	17	11–23
Sohounta	19	15	79	61–97
Za-Agbokpa	12	7	58	30–86
Za-Dokpa	45	24	53	38–68
Za-Kekere	302	170	56	50–62
Za-Kpota Centre	26	15	58	39–77
Za-Zoume	152	84	55	47–63
Total	1165	561	48	45–51

### Vector susceptibility to insecticides

**Fig. 6:**
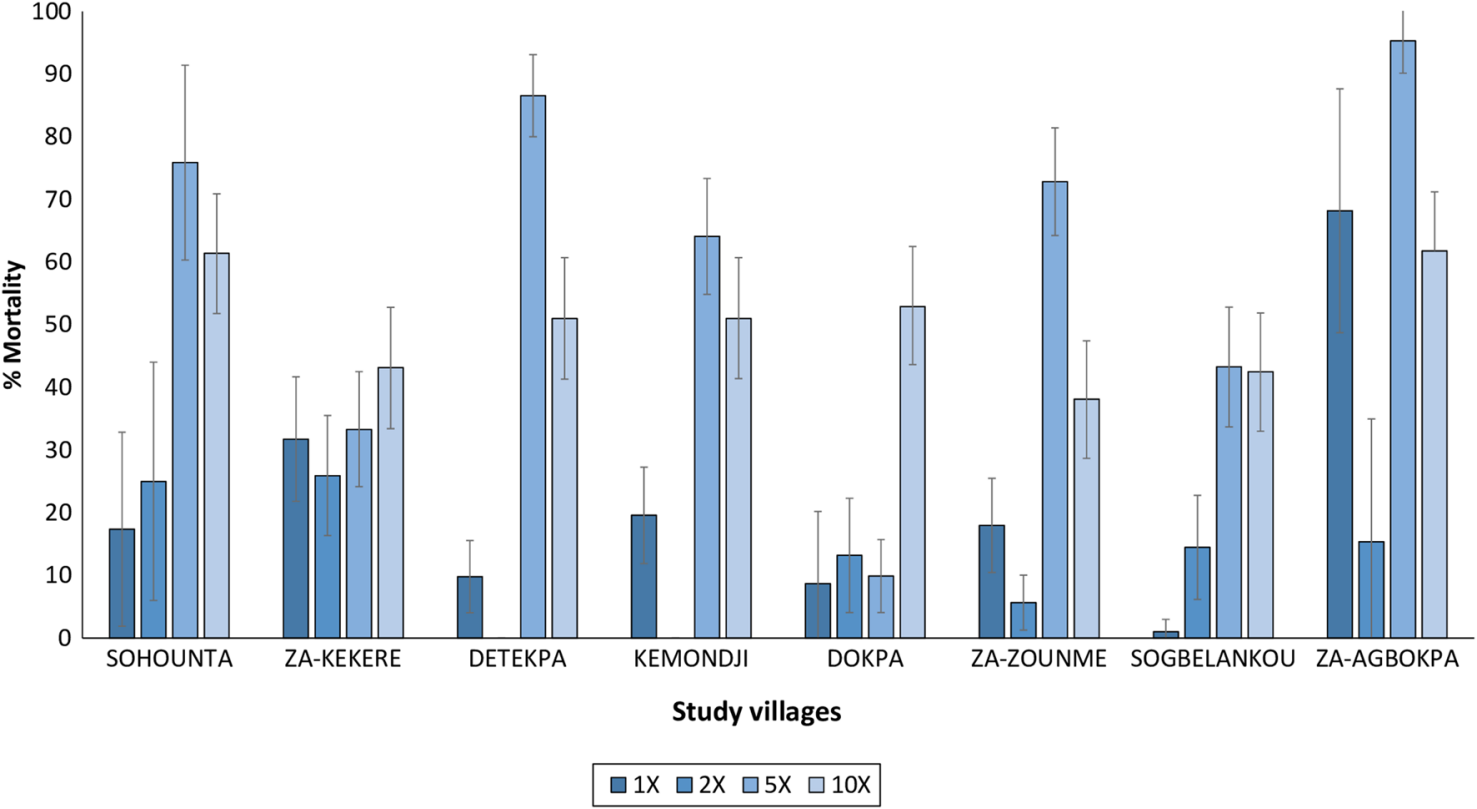
Mortality (24 h) of *An. gambiae* s.l. from eight villages exposed to deltamethrin at 1×, 2×, 5× and 10× the discriminating concentration of 12.5 µg/bottle in bottle bioassays

**Fig. 7:**
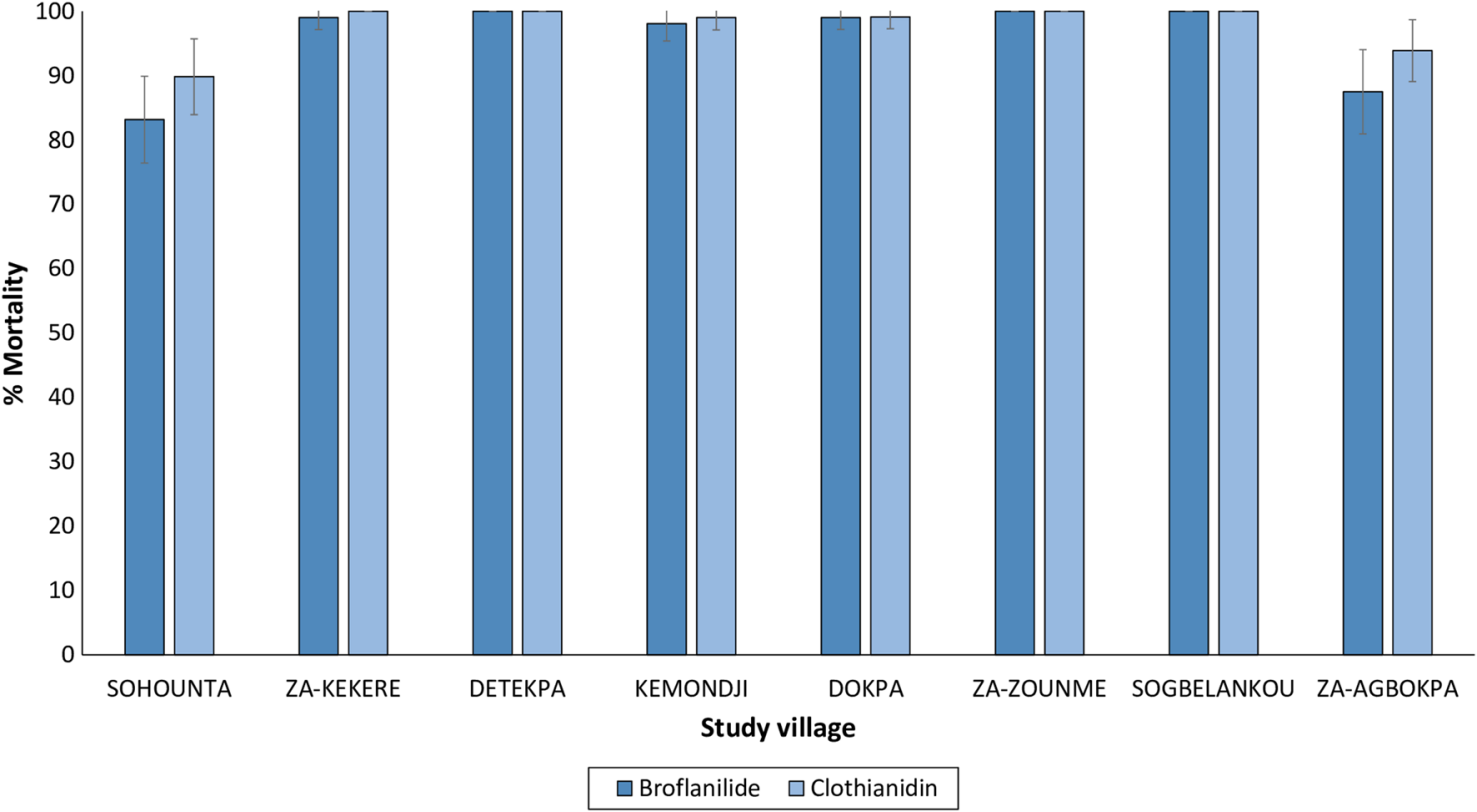
Mortality (72 h) of *An. gambiae* s.l. exposed to broflanilide (6 µg/bottle) and clothianidin (90 µg/bottle) in bottle bioassays

Vector mortality with the discriminating concentration of deltamethrin (12.5 µg/bottle) ranged between 1 and 68% (Fig. 6). Mortality increased at doses 2×, 5× and 10× the discriminating concentration of deltamethrin but did not exceed 98% at 10× in any village, indicating a high intensity of pyrethroid resistance in the study area. With broflanilide tested at 6 µg/bottle, pooled mortality rates across the study villages showed full susceptibility of the vector population in the sub-district to the insecticide (96% mortality). There was however some variability in mortality in broflanilide susceptibility bioassays in two villages (Sohounta and Za-Agbokpa) showing 83% and 88% mortality, respectively (Fig. 7). Sub-optimal mortality in these villages was probably greater due to variability in bioassays with mosquito sub-populations rather than resistance given that the broflanilide susceptibility assay method and the discriminating concentration for broflanilide had not been fully optimised at the time of the baseline study. Subsequent bottle bioassays conducted post-intervention showed full susceptibility to broflanilide in the vector population [45]. The results also showed no evidence of resistance to clothianidin tested at 90 µg/bottle; mortality rates were generally > 90% in all villages. No mortality was recorded in the controls.

### Insecticide resistance genotypes

The total number of *An. gambiae* s.l. mosquitoes analysed for target site insecticide resistance genes was 460 for the L1014F *kdr* mutation and 467 for the G119S *ace-1* mutation. The frequency of L1014F *kdr* in the *An. gambiae* complex across the study area was 84%, while the frequency of the *ace-1* mutation was 26% (26%) (Table 8).

**Table 8:** Frequency of the L1014F *kdr* and G119S *ace-1* mutations across the study area

Mutation	N Tested	RR	RS	SS	Frequency	95% CI
L1014F kdr	460	347	77	36	84	81–87
G119S ace-1	467	14	212	241	26	22–30

## Discussion

The purpose of this study was to collect baseline information on the prevalence and transmission of malaria in the Zakpota Centre sub-district of Benin in preparation for a community randomised trial to evaluate a new insecticide for IRS. First, we found a malaria prevalence of 19% among children under 5, the most vulnerable age group to malaria. This prevalence was lower than the 42% prevalence reported in this age group in a similar larger-scale study performed in the Zou region of Benin in preparation for a randomised controlled trial of dual-AI ITNs [34]. The difference in malaria prevalence between the two studies may be attributed to the timing of the surveys given that the present study was performed at the onset of the main rainy season (April to May) when cases can be low, while the latter was performed during the second rainy season (October to November).

The main vector found in the study area was *An. gambiae* s.l., consisting of both *An. gambiae* s.s. and *An. coluzzii* with the former available in higher overall proportions and at most time points except in the middle of the dry season. This finding confirms several studies that have demonstrated that both species commonly occur in sympatry in West Africa, though they may differ substantially in their ecological behaviour, insecticide resistance status and vectorial capacity [46–48]. Previous entomological surveys in Benin have also shown a wide variability in the proportion of both sibling species in terms of geographical spread and seasons with reports of higher densities of *An. gambiae* s.s. increasing more recently [8, 9, 15, 49]. Apart from *An. gambiae* s.l., no other malaria vector species were found in the study area probably due to the absence of suitable breeding sites for their development.

Vector biting rates were greater indoors compared to outdoors, thus demonstrating the suitability of indoor vector control interventions including IRS for malaria control in the study area. However, the high levels of outdoor biting observed (44%) are nonetheless of concern. This corroborates several studies that have reported high outdoor malaria vector biting rates in Benin [12, 50] and elsewhere [51]. This finding highlights the need for additional vector control interventions that target outdoor biting vectors. Vector biting was highest at night (1:00 a.m. to 5:00 a.m.) both indoors and outdoors which aligns with the well-documented nocturnal behaviour of anopheline mosquitoes [21, 52]. As expected, the biting rates of daytime *Aedes* mosquitoes collected in the study area were substantially lower during this period. The overall annual EIR in the study area across the baseline entomological surveys was 16.1 bites per person with 2% of mosquitoes found to be infected with malaria sporozoites. Modelling studies suggest that this level of EIR would lead to a higher malaria prevalence than the 19% observed in the cross-sectional survey [53]. EIR also varied widely across the villages in the study area which can be explained by a high heterogeneity in anopheline densities and their infectivity across the individual villages.

The high intensity of pyrethroid resistance in susceptibility bioassays in this study aligns with several previous studies performed in the Zou region [15, 21] and Benin at large [17, 20, 23, 24, 54, 55]. The genotyping studies showed high frequencies of the L1014F *kdr* gene (84%) which would have contributed to the levels of phenotypic resistance observed. The lower levels of the G119S *ace-1* (26%) corroborate the relatively lower levels of organophosphate and carbamate resistance observed in the Zou region and in most areas of Benin [20, 56, 57]. By contrast, evidence for resistance to broflanilide and clothianidin was not found, thus demonstrating the suitability of the study area for a trial deploying both insecticides for IRS in the study area.

## Conclusion

This study found high levels of malaria prevalence, vector density and transmission in the Zakpota sub-district, despite the high use of ITNs in the study area. The vector population was mostly indoor resting and showed a high intensity of pyrethroid resistance but was generally susceptible to broflanilide. These findings demonstrated the suitability of the study area for the assessment of a new broflanilide insecticide product (VECTRON™ T500) for IRS in a community randomised trial. However, the levels of outdoor biting observed were high, which demonstrates the need for suitable vector control strategies to target malaria transmission by outdoor biting mosquitoes.

### Supplementary Information

Additional file 1.

## Data Availability

The aggregated datasets used and/or analysed during the current study are provided as supplementary information.

## Abbreviations

AI: Active ingredient
CDC-LT: Centers for Disease Control and Prevention miniature light trap
CHW: Community health worker
CREC: Centre de Recherche Entomologique de Cotonou
CSP: Circumsporozoite protein
EIR: Entomological inoculation rate
HBR: Human biting rate
HLC: Human landing catch
IRR: Incidence rate ratio
IRS: Indoor residual spraying
ITN: Insecticide-treated net
LSHTM: London School of Hygiene & Tropical Medicine
PSC: Pyrethrum spray catch
RDT: Rapid diagnostic test
SR: Sporozoite rate
WHO: World Health Organization
