# Supplementary figures and images for: Malaria prevalence and transmission in the Zakpota sub-district of central Benin: baseline characteristics for a community randomised trial of a new insecticide for indoor residual spraying
Source: Parasit Vectors. 2024 Jul 13;17:303. doi: 10.1186/s13071-024-06342-1 (PMC11245802; doi:10.1186/s13071-024-06342-1)

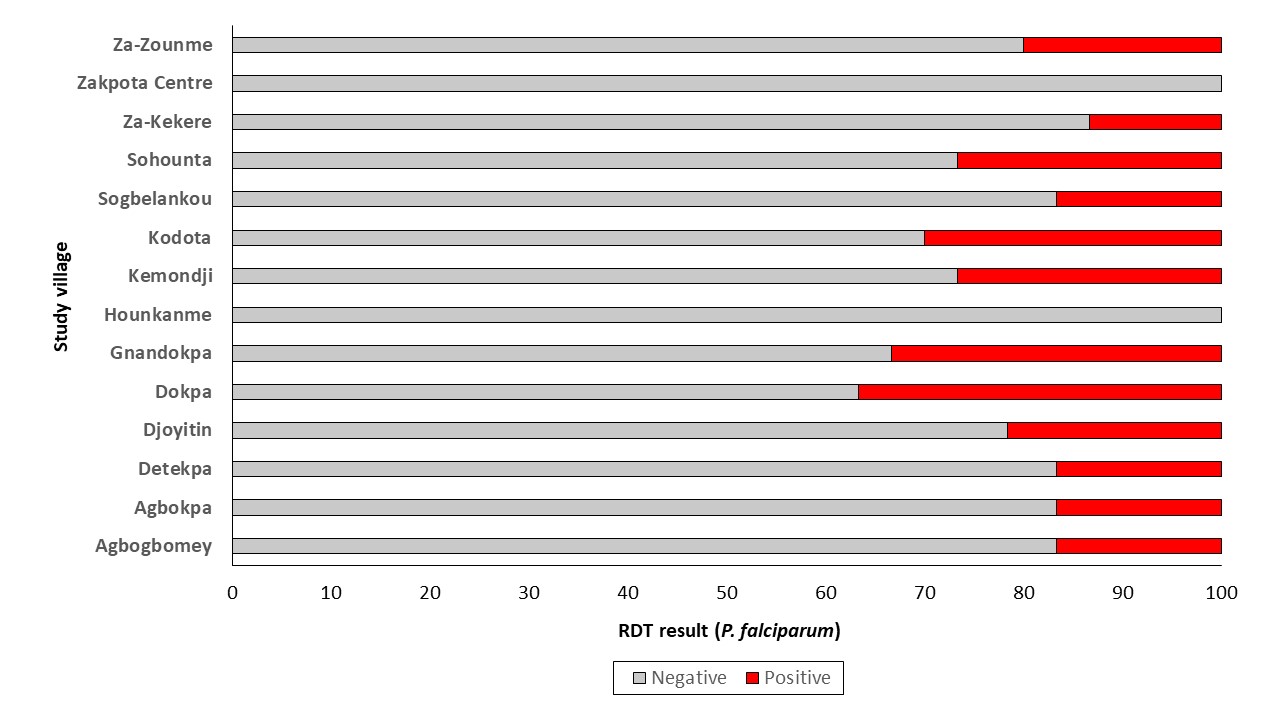

Supplement: Supplementary file 1 — Additional file 1. [file 13071_2024_6342_MOESM1_ESM.jpg]
